# Modern management of third nerve palsy: neuroimaging triage and surgical rehabilitation

**DOI:** 10.3389/fneur.2026.1971851

**Published:** 2026-10-29

**Authors:** Mastour Abdullah Alhamami

**Affiliations:** Department of Optometry, College of Applied Medical Science, King Saud University, Riyadh, Saudi Arabia

**Keywords:** blepharoptosis, computed tomography angiography, diagnostic triage, neuroimaging, oculomotor nerve diseases, ophthalmoplegia, strabismus, third nerve palsy

## Abstract

Third nerve palsy (TNP) is a complex neuro-ophthalmic condition that requires rapid differentiation between benign microvascular ischemia and life-threatening or otherwise serious causes, including aneurysmal, neoplastic, inflammatory, infectious, demyelinating, traumatic, and cavernous sinus pathologies. Given the high prevalence of diabetes, understanding both pupil-involving and pupil-sparing presentations is critical. Current literature often fragments TNP management across isolated medical specialties. Rather than presenting an evidence-based consensus guideline, this paper provides an expert narrative synthesis proposing a bipartite clinical framework. This framework sequences acute neuro-ophthalmic triage (Weeks 1–12) and chronic ophthalmic rehabilitation (>6 months) to coordinate immediate etiological workup with subsequent surgical and rehabilitative planning. TNP manifestations vary based on the dual anatomy of the third nerve. Compressive lesions typically paralyze superficial pupillomotor fibers, whereas ischemic insults preserve them. Because pupillary status alone cannot definitively rule out emergencies, acute care triage requires neuroimaging tailored to the clinical context. Magnetic Resonance Angiography (MRA) and Computed Tomography Angiography (CTA) serve as primary modalities when an aneurysm is suspected; contrast-enhanced MRI is preferred when brainstem, cavernous sinus, orbital, inflammatory, infectious, or neoplastic pathology is suspected; and CT is used first in acute trauma. Digital Subtraction Angiography (DSA) is reserved for specific cases to detect microaneurysms smaller than 3 mm when initial imaging is inconclusive. Chronic or unresolving cases are managed through sequentially planned interventions, where strabismus alignment is typically prioritized before ptosis repair. Ultimately, this review proposes an evidence-informed, phase-based framework designed to support the timely identification of potentially fatal etiologies and guide individualized functional and esthetic rehabilitation. Multidisciplinary collaboration, utilizing prospective registries, is essential to address current data gaps and enhance long-term rehabilitative success in patients with TNP.

## 1 Introduction

The oculomotor nerve (CN III) comprises outer parasympathetic fibers that regulate the pupil and accommodation, and inner somatic fibers that innervate the levator palpebrae superioris (LPS) and four extraocular muscles (1). While complete paralysis of CN III yields a highly recognizable “down and out” positioning of the globe with pronounced ptosis (2, 3), overlapping etiologies heavily complicate third nerve palsy (TNP) diagnosis. Clinicians are frequently forced to rapidly differentiate between benign microvascular events and life-threatening compressive lesions (4). Because the diverse manifestations of this condition can obscure clinical decision-making during critical care windows, historical textbook descriptions often lead to disjointed diagnostic and rehabilitative steps across isolated medical specialties (5, 6).

The clinical presentation diverges significantly depending on whether the underlying etiology is a compressive lesion (e.g., posterior communicating artery (PComA) aneurysms, trauma, or uncal herniation) or an ischemic lesion (most commonly secondary to diabetes mellitus or hypertension) (7, 8). Despite classical clinical distinctions, relying solely on pupillary status is insufficient to safely rule out life-threatening emergencies (5, 9). Painful TNP with pupil sparing in diabetic patients often indicates a microvascular ischemic event, but failure of the palsy to resolve or worsening pain necessitates urgent re-evaluation (9–11). Rapid clinical evaluation and urgent neuroimaging tailored to the clinical context are paramount to identify aneurysmal and other serious causes, including vascular malformations such as carotid-cavernous fistulas (CCFs), as well as central ischemic, neoplastic, inflammatory, infectious, demyelinating, and traumatic lesions (12–18).

Recent literature has been instrumental in determining clinical outcomes for specific neurosurgical and diagnostic profiles (17, 19, 20). However, because these highly specialized studies focus narrowly on isolated therapeutic or diagnostic questions, the resulting data remains heavily fragmented across the distinct silos of the neurology, neurosurgery, and ophthalmology literature. To resolve these clinical ambiguities and bridge the gap between disciplines, our expert narrative synthesis translates existing evidence into a cohesive, timeline-driven treatment framework. Rather than presenting new primary clinical data, this bipartite roadmap structures historically isolated evidence into two operational phases:

Phase 1 (Weeks 1–12): Details immediate diagnostic triage (Days 1–7) to safely evaluate acute manifestations and identify aneurysmal and other serious causative pathology, followed by a conservative observation window for patients in whom a presumed microvascular palsy is supported.

Phase 2 (> 6 Months): Critically assesses advanced, muscle-specific surgical advancements (such as sophisticated transposition and ptosis correction methods) alongside non-surgical neurorehabilitation interventions for attaining functional and esthetic alignment in persistent deficits.

## 2 Methods of literature search

A comprehensive narrative review of the PubMed and MEDLINE databases was conducted for literature published up until July 2026. The primary objective was to synthesize current diagnostic and surgical evidence to construct a multidisciplinary, timeline-driven clinical pathway for unilateral TNP. The search approach was specifically designed to capture contemporary diagnostic and surgical breakthroughs, prioritizing literature published within the last 15 years to ensure the proposed clinical pathways reflect modern management paradigms. However, select foundational publications from the early 2000s were retained specifically to provide necessary context for established strabismus and ptosis techniques.

The core search strategy utilized combinations of the following terms: “third nerve palsy,” “diabetic third nerve,” “oculomotor nerve palsy,” “isolated oculomotor nerve palsy,” “oculomotor nerve paralysis,” “oculomotor nerve palsy treatment,” “isolated third nerve,” and “isolated third nerve palsy.” These terms were iteratively combined with specific concept terms representing phase-specific interventions (e.g., “CTA,” “MRA,” “muscle transposition,” “Botulinum Toxin”) to retrieve literature relevant to each distinct phase of care.

Inclusion criteria were restricted to articles in English that focused on adult or pediatric populations and provided actionable data on neuroimaging techniques or surgical results. While adult cases are predominantly driven by microvascular or aneurysmal etiologies (21), pediatric TNP literature highlights different primary etiologies (congenital, traumatic, or inflammatory) and prioritizes amblyopia risk management (22). To ensure the statistical reliability of reported surgical and rehabilitative outcomes, studies with fewer than five patients were generally omitted from our review. However, targeted exceptions were made for high-impact case reports detailing rare, life-threatening neurovascular presentations that directly informed emergency triage protocols.

Study selection and critical appraisal were expert-driven, prioritizing high-impact publications, clinical guidelines, and studies that directly addressed protocols or outcomes related to the stated objectives. Because this manuscript is designed as a narrative review synthesizing current diagnostic and surgical evidence, rather than a formal systematic review or meta-analysis—a PRISMA (Preferred Reporting Items for Systematic Reviews and Meta-Analyses) flowchart for paper stratification is not methodologically applicable.

### 2.1 Pathophysiology and clinical manifestations

CN III can suffer damage anywhere along its five distinct neuroanatomical segments: the midbrain nucleus, midbrain fascicles, subarachnoid space, cavernous sinus, and orbit (2, 23). The clinical features are tightly bound to the precise site of injury and the nature of the lesion, as outlined in Table 1.

**Table 1:** Anatomical localization, clinical presentation, and triage of TNP.

Anatomical segment	Lesion classification	Primary etiologies	Key clinical presentation	Pupillary involvement	Red flags	Recommended neuroimaging	Differential diagnoses
Midbrain nucleus	Nuclear lesion	Ischemia, neoplasm, demyelination	Bilateral ptosis; contralateral elevation deficit	Rarely affected	Bilateral neurological signs	MRI Brain with and without contrast (DWI for ischemia)	Ischemic stroke, demyelinating disease, midbrain neoplasm
Midbrain fascicle	Fascicular lesion	Ischemic infarction, hemorrhage	Neurologic deficits combined with contralateral hemiplegia	Variable	Hemiparesis, ataxia	MRI Brain with and without contrast (DWI for ischemia)	Weber syndrome, Claude syndrome, Benedikt syndrome
Subarachnoid space	Compressive	PComA aneurysm	Fixed, dilated pupil; complete external ophthalmoplegia	Present (Involved)	Severe acute headache, altered mental status	Urgent CTA or MRA; DSA for select cases if initial imaging is inconclusive	PComA aneurysm compression, uncal herniation, infectious/malignant meningitis
Subarachnoid space	Ischemic microangiopathy	Diabetes mellitus, hypertension	Isolated strabismus; preserved pupillary light reflex	Spared	Worsening pain, no improvement at 4–6 weeks	Close observation in classic cases; urgent neuroimaging (MRI/MRA or CTA) if atypical features, red flags, progression, or non-resolution occur	Microvascular ischemia, evolving compressive lesion
Cavernous sinus	Multineuronal syndrome	Tolosa-Hunt syndrome, CCF	Painful ophthalmoplegia; multiple cranial nerve deficits	Variable	Proptosis, chemosis, multiple CN involvement	MRI Brain and Orbits with contrast; CTV or MRV if venous thrombosis is suspected	Carotid-cavernous fistula (CCF), cavernous sinus thrombosis, Tolosa-Hunt syndrome, pituitary apoplexy
Orbit	Localized orbital lesion	Trauma, localized inflammation	Partial, division-specific palsies; localized proptosis	Variable	History of trauma, severe proptosis	MRI or CT Orbits with contrast	Orbital apex syndrome, orbital cellulitis, severe thyroid eye disease, orbital trauma/fracture

Distinguishing between adult and pediatric populations is vital when evaluating the underlying etiology of a TNP, as their clinical profiles dictate divergent diagnostic approaches. While adult presentations are overwhelmingly dominated by microvascular ischemia or compressive lesions, pediatric cases are distinctly different. In children, congenital anomalies, birth trauma, closed head injuries, and post-viral inflammatory conditions represent the most common etiologies, whereas aneurysmal compression or diabetic microvascular ischemia is exceedingly rare (6, 24). The diagnostic algorithm for pediatric patients shifts away from acute vascular screening and instead prioritizes broad-spectrum neuroimaging, specifically favoring MRI to minimize the risk of ionizing radiation exposure while ruling out structural, neoplastic, or inflammatory causes (25, 26).

### 2.2 The pupil rule: microvascular ischemia vs. structural compression

The structural organization of the third nerve explains the crucial clinical distinction between pupil-involving and pupil-sparing palsies (1). The small, unmyelinated parasympathetic pupillomotor fibers run superficially along the outer dorsomedial aspect of the nerve sheath (1). The blood supply to these fibers is sourced from the adjacent pial network (1). Extrinsic mechanical forces—such as an expanding PComA aneurysm, trauma, or uncal herniation—directly compress these outer fibers first, leading to an unresponsive, dilated pupil and loss of accommodation (23, 27).

Conversely, the inner somatic trunk is supplied by the microvascular network of the vasa nervorum. Microvascular diseases, such as diabetes mellitus and hypertension provoke deep ischemic necrosis within the central core of the nerve (1). This inner core damage spares the outer parasympathetic fibers, leaving pupillary function intact and reactive to light (17). In adult patients over the age of 50, an isolated, pupil-sparing TNP is most often secondary to diabetes mellitus or hypertension (24, 28, 29).

### 2.3 Sensory symptoms and aberrant regeneration

Patients with TNP typically report acute binocular diplopia due to eccentric image placement on the extrafoveal retina (30). Paradoxically, severe ptosis often hides this symptom by acting as a natural occluder. Ocular pain is an important warning feature (3, 6). Severe, sudden-onset, progressive, or atypical headache or orbital pain should lower the threshold for urgent neuroimaging, particularly when accompanied by pupillary involvement, neurological deficits, proptosis, chemosis, or other atypical features. However, pain may also occur in microvascular ocular motor nerve palsy and should not be used as an isolated etiological discriminator (22, 31). When compressive or traumatic injuries disrupt the endoneurial sheath, misdirected axonal regrowth can lead to aberrant regeneration (32). This causes lid-gaze dyskinesis (the pseudo-Graefe sign, where the upper lid retracts during adduction) or pupil-gaze dyskinesis, resulting in paradoxical pupillary constriction during convergence or downward gaze (25, 31, 32).

### 2.4 Phase 1: acute management and diagnostic triage (weeks 1–12)

While Phase 1 covers the first 12 weeks, clinical urgency is dictated by etiological suspicion. We categorize the first 7 days as “Immediate Emergency Triage,” requiring rapid exclusion of life-threatening compressive (aneurysmal), inflammatory or infectious (e.g., GCA), neoplastic, or traumatic etiologies (18). The subsequent period (Weeks 2–12) functions as “Recovery Monitoring” for patients in whom no causative abnormality has been identified and the presentation is consistent with microvascular palsy, whether neuroimaging was negative or was appropriately deferred in a classic presentation (18, 28). Patients who do not meet these criteria are not observed; they proceed to specialist review and further targeted evaluation. Figure 1 summarizes this pathway, a general acute diagnostic triage and management pathway for adult third nerve palsy that includes aneurysmal and nonaneurysmal causes.

**Figure 1:**
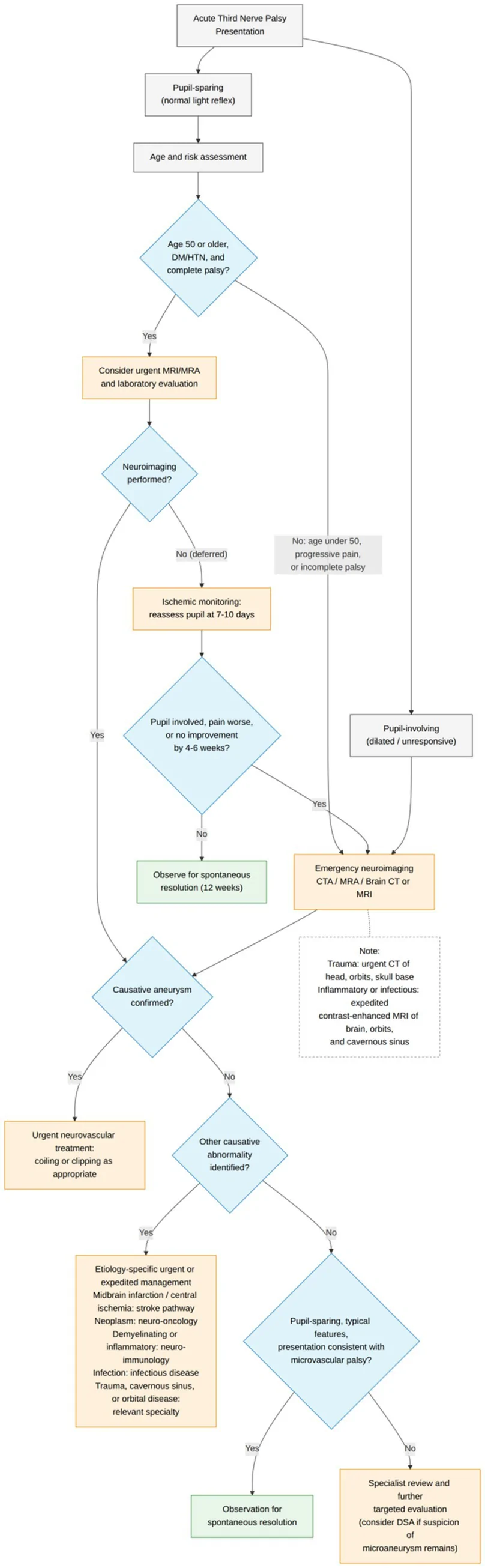
General acute diagnostic triage and management pathway for adult third nerve palsy, covering both aneurysmal and nonaneurysmal causes and not limited to the distinction between presumed microvascular palsy and aneurysmal compression. Emergency neuroimaging is tailored to the presentation: CTA or MRA for suspected aneurysm; CT of the head, orbits, and skull base in acute trauma; and contrast-enhanced MRI of the brain, orbits, and cavernous sinus for suspected inflammatory, infectious, neoplastic, demyelinating, brainstem, cavernous sinus, or orbital pathology. In pupil-sparing patients aged 50 years or older with DM or HTN and a complete palsy, imaging may be deferred, with reassessment at 7–10 days. If a causativeaneurysm is confirmed, urgent neurovascular treatment (coiling or clipping, as appropriate) is indicated. If not, management is guided by other imaging findings and the clinical context, and midbrain infarction or other central ischemia, neoplastic, demyelinating, inflammatory, infectious, traumatic, cavernous sinus, or orbital lesions are managed by the relevant specialty. Observation is reserved for patients with typical features and no causative abnormality; otherwise, specialist review and further targeted evaluation follow, with DSA considered if suspicion of a microaneurysm remains. CT, computed tomography; CTA, CT angiography; DM, diabetes mellitus; DSA, digital subtraction angiography; HTN, hypertension; MRA, magnetic resonance angiography; MRI, magnetic resonance imaging.

The acute management window requires prioritizing this rapid diagnostic triage over surgical corrections to identify life-threatening neurovascular and other serious conditions (18). When a patient presents with ophthalmoplegia, ptosis, and mydriasis, immediate neuroimaging is required to rule out compressive lesions, which can be fatal (18, 26). As shown in Figure 1, pupil-sparing patients under 50 years, or with progressive pain or an incomplete palsy, also go directly to urgent neuroimaging, and the imaging result then determines management as described in Step 3.

#### 2.4.1 Step 1: baseline ocular and systemic examination

The initial clinical assessment focuses on defining the extent of the oculomotor deficit. Clinicians must perform cover testing across all nine diagnostic positions of gaze to quantify the strabismus, which typically manifests as a classic exotropia and hypotropia (5–7). This assessment determines whether the palsy is complete or partial; atypical, incomplete presentations can indicate an evolving compressive lesion and warrant a lower threshold for urgent neuroimaging. Simultaneously, the upper eyelid should be examined to assess whether the ptosis fully obscures the visual axis (6–8). For suspected microvascular cases, it is essential to immediately record systemic blood pressure and order baseline laboratory evaluations, including a complete blood count (CBC), hemoglobin A1c (HbA1c), C-reactive protein (CRP), and erythrocyte sedimentation rate (ESR) (2, 6). Giant cell arteritis (GCA) should be considered in older patients with compatible systemic symptoms, elevated inflammatory markers, visual symptoms, or ischemic features (1, 17). Although isolated third nerve palsy is an uncommon manifestation, its recognition is important because delayed diagnosis may result in irreversible visual loss and other ischemic complications (2, 18). Depending on the presentation, neuroimaging should be considered concurrently to exclude aneurysmal and other structural causes (18, 28).

#### 2.4.2 Step 2: neuroimaging protocol selection

Advanced imaging must be selected based on pupillary status, pain characteristics, completeness of the presentation, age, vascular risk factors, progression of the deficit, associated neurological or orbital signs, and any traumatic, inflammatory, or infectious context (18, 26), as summarized in the triage algorithm (Figure 1). Practical decision-making for selecting the appropriate modality depends heavily on the suspected etiology and specific clinical subtype. Magnetic Resonance Imaging (MRI) serves as the standard for direct cranial nerve and soft-tissue evaluation (18, 26). T1, T2, and Diffusion-Weighted Imaging (DWI) sequences are indispensable for detecting brainstem infarctions, demyelination, or neoplasms (18, 26). High-resolution MRI with contrast, specifically targeting the brain, orbits, and cavernous sinus, is strongly recommended when the patient presents with multiple cranial neuropathies or orbital signs (e.g., chemosis, proptosis) indicating a cavernous sinus or orbital apex syndrome (18). MRI is the preferred frontline modality for pediatric TNP to limit radiation exposure, and for recurrent TNP to rule out slow-growing tumors or chronic inflammatory processes (21, 25). At the subarachnoid level, time-of-flight Magnetic Resonance Angiography (MRA) provides specialized, non-invasive vascular mapping (18, 22, 26).

Conversely, non-contrast brain CT is the immediate choice in emergency settings to rapidly rule out acute subarachnoid hemorrhage or uncal herniation, and it is the initial imaging of choice for traumatic TNP to assess for orbital or skull base fractures (31, 32). Computed Tomography Angiography (CTA) features high spatial resolution and offers up to 90% sensitivity for detecting cranial aneurysms 3 mm or larger (33, 34). CTA is often preferred over MRA for rapid emergency vascular screening and is the ideal alternative for individuals with claustrophobia or metallic implants (33, 34).

The clinical context influences the urgency, modality, and anatomical areas of interest for neuroimaging. In acute trauma, urgent CT of the head, orbits, and skull base is indicated to rule out fractures and intracranial injury, with CT angiography or MRI added as guided by the suspected injury pattern (e.g., suspected vascular injury, cavernous sinus involvement, or brainstem injury) (26, 33). If an inflammatory, infectious, or neoplastic process is suspected, particularly in the presence of cranial neuropathies, fever or systemic inflammatory symptoms, immunosuppression, meningism, or orbital signs suggesting cavernous sinus involvement, urgent contrast-enhanced MRI of the brain, orbits, and cavernous sinus is recommended, with anatomical coverage guided by the suspected site of pathology (18, 33).

Catheter-based Digital Subtraction Angiography (DSA) is reserved for highly specific cases, acting as the definitive diagnostic standard for detecting tiny microaneurysms smaller than 3 mm or low-flow dural-cavernous fistulas (21, 34, 35). Because it carries an inherent risk of stroke and is highly invasive, DSA is typically reserved for cases when initial non-invasive CTA or MRA is negative, but the patient presents with a painful, pupil-involving TNP, or an atypical partial (incomplete) TNP, where the clinical suspicion for a microaneurysm remains high (19, 33–35).

For pupil-sparing presentations, contemporary imaging thresholds must be strictly applied. Historically, pupil-sparing patients 50 years of age or older with vascular risk factors were managed solely with laboratory evaluation and observation (9, 28). However, because aneurysms can occasionally present without initial pupillary involvement, some contemporary clinical paradigms suggest considering urgent high-resolution imaging (such as MRI or MRA) even in this older demographic to rule out structural compression, though this aggressive screening approach remains actively debated in routine clinical practice (17, 18, 25). If initial imaging is deferred in favor of observation—typically for patients presenting with an isolated, complete, painless, and pupil-sparing TNP who fit the classic microvascular demographic—clinicians must adhere to an explicit threshold for emergency re-evaluation. Urgent neuroimaging is triggered if the pupil becomes involved at the 7–10 day reevaluation, if pain worsens, if motor symptoms fail to improve within 4 to 6 weeks, or if atypical features (e.g., aberrant regeneration) develop (2, 23, 31).

#### 2.4.3 Step 3: etiology-directed management, medical stabilization, and observation

After urgent neuroimaging, management follows Figure 1: urgent neurovascular treatment, including endovascular coiling or microsurgical clipping as appropriate, if a causative aneurysm is confirmed (13, 18, 19); etiology-specific management if another causative abnormality is identified, such as a stroke pathway for midbrain infarction or other central ischemia, neuro-oncology for neoplasm, neuro-immunology for demyelinating or inflammatory disease, infectious disease management for infection, and the relevant trauma, cavernous sinus, or orbital specialty (18, 22, 33); and specialist review with further targeted evaluation, including DSA if suspicion of a microaneurysm remains, when the presentation is atypical (18, 28, 34, 35).

For patients who reach observation, If no causative abnormality is identified and the presentation is consistent with microvascular palsy, whether neuroimaging was negative or was appropriately deferred in a classic presentation, management shifts toward medical stabilization. Microvascular ischemic palsies follow a self-limiting course, and most cases resolve spontaneously within 6 to 12 weeks (4). However, recent meta-analyses demonstrate that isolated ischemic oculomotor nerve palsies are not entirely benign; patients with an ischemic TNP face up to an eightfold increased risk of subsequent stroke within the first year following the palsy (36). Acute care during this observation window must center on internal medicine optimization—specifically stringent glycemic control and blood pressure regulation—paired with symptomatic diplopia relief to mitigate long-term systemic vascular risks (10, 11, 36).

### 2.5 Phase 2: chronic management and interventions (>6 months)

Surgical management is generally deferred until the clinical presentation has remained stable and unresolved for at least 6 months, allowing for the possibility of spontaneous microvascular recovery (37). While a systematic strategy in which strabismus correction precedes ptosis repair is typically recommended—since vertical extraocular muscle adjustments can significantly alter the resting position of the upper eyelid—this sequencing requires careful clinical nuance (38). Important exceptions to this timeline exist. For instance, severe corneal exposure may necessitate earlier, temporizing eyelid intervention, while in pediatric populations, immediate visual axis obstruction poses a profound amblyopia risk that supersedes standard surgical timelines and demands urgent ptosis correction (22, 39, 40). Patient age, cosmetic concerns, and the psychosocial burden of the palsy must also be weighed heavily when timing these sequential interventions. Current literature indicates that while achieving a functional field of single binocular vision in the primary gaze is a realistic goal, complete restoration of physiological motility is rarely attainable (38, 41). A 2024 prospective analysis confirmed that tailored extraocular muscle transpositions successfully collapse complex strabismus patterns and improve primary position alignment even in profound CN III deficits, provided patient selection is rigorous (42). Tailored muscle transposition procedures for partial palsies generally report alignment success rates between 60 and 80%, though definitive outcomes vary greatly depending on the extent of native muscle preservation and follow-up duration, and must be interpreted cautiously due to the retrospective and heterogeneous nature of the supporting literature (20, 42, 43). Conversely, severe complete palsies requiring supramaximal resections or mechanical globe fixation carry higher risks of consecutive deviations, often requiring intensive, long-term monitoring (37, 41). Across all major surgical approaches, the risk of residual peripheral diplopia and exposure keratopathy must be thoroughly discussed with patients preoperatively. The frequent need for subsequent reoperations—with reported revision rates often exceeding 30% specifically within heterogeneous cohorts managing complete palsies, traumatic etiologies, or those requiring complex supramaximal resections—highlights the necessity of establishing realistic rehabilitative expectations (20, 37, 41).

#### 2.5.1 Section A: non-surgical and conservative temporizing measures

Before proceeding to permanent surgery, several temporary strategies can assist with vision rehabilitation. Initially, alternate occlusion therapy (patching) the dominant eye or using frosted lenses mitigates intractable diplopia. This is the standard of care to prevent amblyopia development, particularly in pediatric populations, provided all refractive errors are simultaneously corrected (21, 25). Additionally, temporary Fresnel prisms or ground-in prisms are integrated into prescription glasses to realign small horizontal or vertical deviations. This helps patients achieve binocular fusion in the primary gaze position once the ptotic lid begins to elevate (21, 25). For active deviation management, injecting botulinum toxin directly into the functional, unopposed lateral rectus muscle induces temporary paralysis. This safely treats exotropia and counters horizontal drift, positioning the eyes in the primary position while awaiting spontaneous nerve regeneration during the late observation period (38).

Conservative management differs significantly between adult and pediatric cohorts. In adult patients, conservative measures such as unilateral patching, Bangerter foils, or Fresnel prisms are utilized primarily for the symptomatic relief of intractable diplopia while awaiting spontaneous recovery (25, 30). Conversely, in pediatric patients—particularly those within the critical period of visual development under the age of eight—the overarching clinical priority is the rapid prevention of stimulus deprivation amblyopia (6, 38). If a pediatric ptosis completely obstructs the visual axis, or if severe strabismus disrupts binocular fusion, clinicians cannot simply observe the patient. They must urgently initiate aggressive amblyopia therapy, which typically requires cycloplegic refraction and rigorous patching regimens, independent of the timeline for eventual surgical rehabilitation (38).

#### 2.5.2 Section B: chronic strabismus realignment

When muscle deficits persist past 6 months, strabismus surgery is prioritized to center the globe in the primary position. Restoring full physiological motility is rarely achievable; the primary goal is maximizing the field of single binocular vision (38). The surgical pathways are dictated by the severity of the palsy (Figure 2).

**Figure 2:**
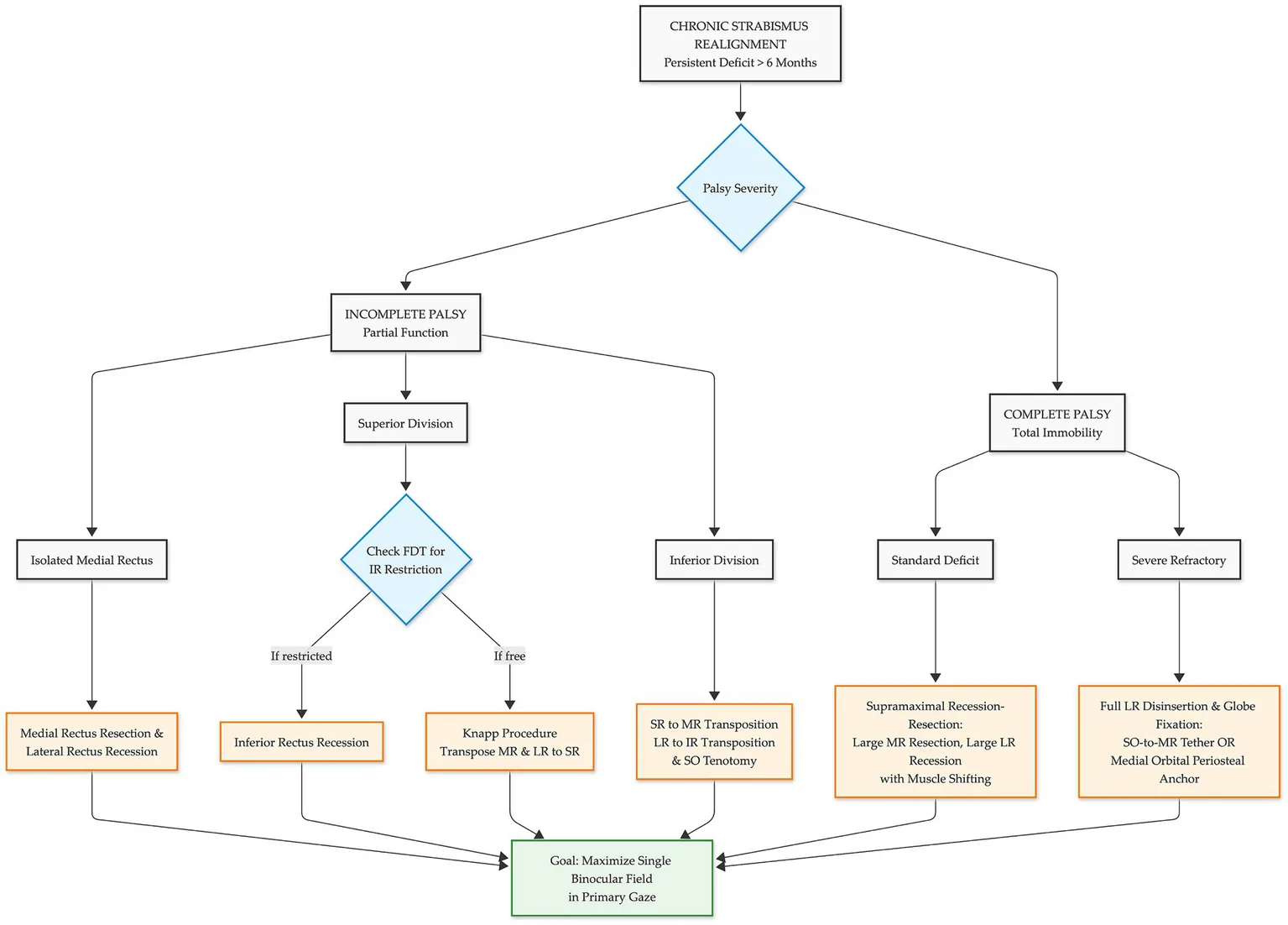
Surgical decision matrix for chronic strabismus realignment. A logical branching hierarchy detailing muscle-specific surgical interventions for persistent deficits lasting beyond 6 months. The pathway bifurcates based on palsy severity. Complete palsies are addressed with supramaximal resections or mechanical globe fixation. Incomplete palsies require highly tailored interventions, such as isolated recess-resect procedures, Knapp transpositions for superior division deficits, or multi-muscle transpositions for inferior division deficits, with the ultimate goal of maximizing the single binocular field in the primary gaze.

In cases of complete TNP affecting all four extraocular muscles, standard muscle adjustments are typically insufficient and surgical goals are limited to achieving alignment in primary gaze. For severe exotropia requiring extensive correction, the lateral rectus can be fully disinserted from the globe and anchored directly to the lateral orbital wall to prevent outward drifting (43). Alternatively, a supramaximal recession-resection may be employed, where a maximal lateral rectus recession is paired with a large medial rectus resection. To correct associated vertical misalignment in this scenario, the resected medial rectus tendon can be shifted upward (supraplacement) or downward (infraplacement) (20, 41). When no active muscle force remains, the eye must be mechanically anchored in place through globe fixation techniques. One established method involves transposing the superior oblique tendon to the medial rectus insertion site to act as a nasal tether (20, 41). Alternatively, the globe can be anchored directly to the medial orbital periosteum using non-absorbable sutures passed near the anterior lacrimal crest (20, 41). While these fixation methods effectively center the eye in the primary gaze, they result in a permanently “frozen globe” with no residual natural mobility, which can sometimes be cosmetically undesirable.

Conversely, for incomplete TNP, surgical plans must be highly tailored to target only the specific paretic muscles identified during testing. Isolated medial rectus involvement is addressed by combining a medial rectus resection with an antagonist lateral rectus recession (20, 41, 43). If the patient presents with superior division palsy, characterized by isolated deficits in superior rectus and LPS function causing impaired elevation, Knapp’s procedure is indicated (20, 41, 43). In this technique, the full tendons of the medial and lateral recti are transposed directly to the superior rectus insertion site (20, 41, 43). Crucially, this is only performed if a Forced Duction Test (FDT) confirms there is no mechanical restriction of the inferior rectus. If inferior rectus restriction is present, a 5–6 mm recession of the inferior rectus must be performed first, ensuring careful dissection of the lower lid retractors to prevent postoperative eyelid malposition (20, 41, 43). For inferior division palsy, multi-muscle strategies are recommended to achieve primary position alignment. This is addressed by transposing the superior rectus tendon to the medial rectus insertion area and the lateral rectus to the inferior rectus insertion site, combined with a superior oblique tenotomy (20, 41). This transposition produces sufficient active force to stabilize the globe and counteracts the intorsion caused by the unopposed action.

#### 2.5.3 Section C: chronic ptosis correction

Ptosis surgery is primarily performed to prevent amblyopia progression and clear the visual axis. Ptosis repair should generally be prioritized after strabismus surgery is complete, as vertical extraocular muscle adjustments can significantly alter the resting position of the upper eyelid. The subsequent surgical approach for eyelid restoration is then dictated by the patient’s residual levator muscle function (Figure 3). For severe ptosis presentations featuring poor native levator muscle function (excursion measurement less than 4 mm), a frontalis sling is the preferred method. In this procedure, a sling material connects the upper tarsal plate directly to the frontalis muscle of the forehead, allowing the patient to open the eye by elevating their brow (39, 40). In contrast, a levator advancement is utilized when the patient retains fair-to-good levator function (excursion measurement 5 mm or greater). Here, the surgeon shortens and tightens the levator aponeurosis by advancing it onto the anterior surface of the tarsus, directly enhancing the muscle’s native lifting capacity (39, 40). Regardless of the approach, surgeons must carefully balance the risk of undercorrection (permanent eyelid retraction) against overcorrection. Overcorrection can lead to exposure keratopathy if the patient cannot close the eyelid completely (39). Due to these intrinsic complexities, subsequent revision operations are frequently required to optimize the final functional outcome. Table 2 summarizes the key diagnostic and therapeutic evidence underpinning this framework.

**Figure 3:**
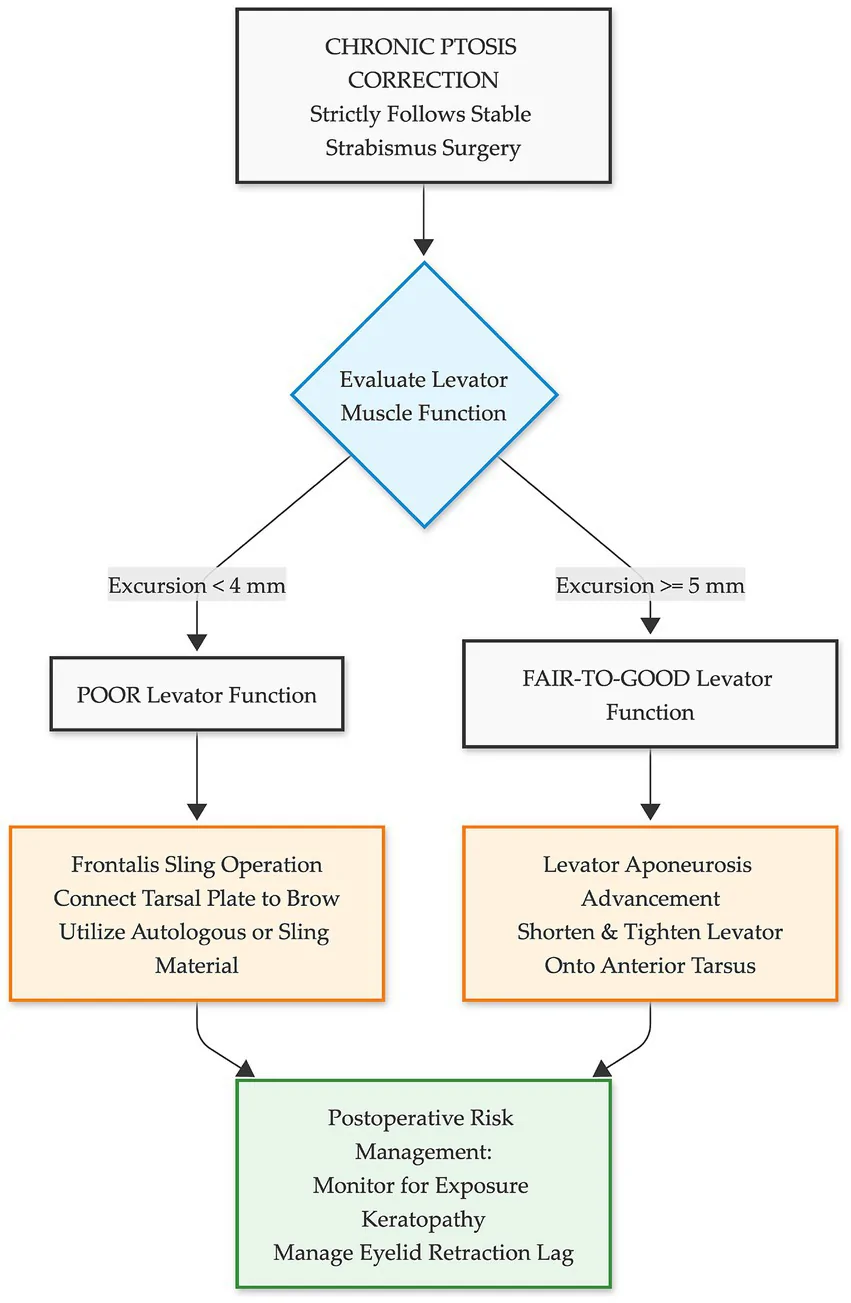
Surgical decision matrix for chronic ptosis correction. A functional flowchart outlining the clinical requirements for eyelid restoration. To prevent severe postoperative lid height malposition, ptosis repair is typically deferred until structural ocular alignment (strabismus surgery) is stable. Surgical selection is dictated by native levator muscle excursion: poor function (<4 mm) requires a frontalis sling operation, whereas fair-to-good function (≥5 mm) allows for a localized levator aponeurosis advancement. Both pathways require vigilant postoperative monitoring to prevent exposure keratopathy and manage potential eyelid retraction.

**Table 2:** Summary of representative key evidence underpinning the clinical framework.

Study (Ref)	Design and population (*N*)	Primary etiologies evaluated	Intervention/Modality evaluated	Main outcomes/Findings	Key limitations
Fang et al. (24)	Retrospective, population-based (*N* = 145)	Microvascular (42%), Neoplasm (11%), Aneurysm (6%)	Diagnostic epidemiology	Microvascular ischemia remains the most common cause of TNP in adults.	Retrospective design; specific geographic population may limit broad generalization.
Jacobson (9)	Prospective observational (*N* = 24)	Pupil-sparing TNP (Microvascular vs. Compressive)	Diagnostic triage; threshold for neuroimaging	Among 24 patients with neurologically isolated relative pupil-sparing TNP, clinical features did not reliably distinguish infarction from mass lesions; neuroimaging was recommended	Small cohort size; predates modern high-resolution CTA/MRA protocols.
Tamhankar et al. (28)	Prospective observational (*N* = 109)	Ischemic vs. Compressive/Other causes	Diagnostic evaluation of presumed microvascular palsies	Found that a significant minority of older patients with presumed microvascular palsies actually had other severe causes, driving the paradigm shift for urgent MRI.	Older prospective cohort (2013); predates the widespread availability of ultra-high-resolution imaging.
Yang et al. (34)	Retrospective cohort (*N* = 1,366)	Intracranial aneurysms	CTA vs. DSA for microaneurysm detection	CTA demonstrated ~90% sensitivity for aneurysms ≥3 mm but poor sensitivity for <3 mm. DSA remains the gold standard for microaneurysms.	Retrospective nature; potential for selection bias regarding which patients underwent DSA.
Shew et al. (36)	Systematic review and meta-analysis (*N* = 2,756 OCNP cases)	Ischemic/Microvascular TNP	Stroke Risk Assessment	Identified an eightfold increased risk of future stroke within the first year following an ischemic TNP.	Relies on historical coding data; varying diagnostic criteria across included studies.
Yahalom et al. (38)	Retrospective review (*N* = 31)	Oculomotor nerve palsy (Complete vs. Partial)	Conservative (Botulinum toxin) & Surgical alignment	Highlighted the utility of Botulinum toxin in the recovery phase, and confirmed partial palsies achieve better surgical binocular fields.	Retrospective design; lack of standardized postoperative diplopia grading metrics.
Merino et al. (37)	Retrospective interventional (*N* = 25)	Traumatic, Ischemic, Congenital	Strabismus surgery (various recess/resect and transpositions)	68% achieved success (deviation <10 PD) after a mean of 1.4 surgeries. Emphasizes the frequent need for reoperation.	Small sample size; heterogeneous surgical approaches limit direct technique comparison.
Ramesh et al. (42)	Prospective interventional (*N* = 20)	Paralytic strabismus including TNP	Extraocular muscle transposition surgeries	Demonstrated significant improvement in primary position alignment and collapse of alphabetical patterns using targeted transpositions.	Single-center cohort; limited sample specifically for isolated TNP.
Bagheri et al. (39)	Retrospective interventional (*N* = 18)	TNP-associated Blepharoptosis	Frontalis sling vs. Levator advancement	Frontalis sling was highly effective for severe ptosis (<4 mm function); highlighted the ongoing risk of exposure keratopathy post-operatively.	Very small cohort; limited long-term follow-up for sling material longevity.

## 3 Discussion

The management of TNP remains one of the most challenging paradigms in neuro-ophthalmology and requires a dual approach that transitions from acute neuro-ophthalmic triage to structured, chronic visual rehabilitation. Quickly and accurately differentiating pupil-sparing microvascular conditions from pupil-involving compressive etiologies remains a vital clinical skill, although pupillary status alone cannot exclude a serious cause (5, 9). While traditional clinical markers generate initial suspicion, neuroimaging tailored to the clinical context is central to identifying aneurysmal and other serious structural causes (18, 26): CTA or MRA for suspected aneurysm, CT of the head, orbits, and skull base in acute trauma, and contrast-enhanced MRI of the brain, orbits, and cavernous sinus for suspected inflammatory, infectious, neoplastic, demyelinating, brainstem, cavernous sinus, or orbital pathology. DSA remains the reference standard for small aneurysms (34, 35). In the proposed pathway (Figure 1), management after imaging depends on whether a causative aneurysm is confirmed and, if not, on other imaging findings and the clinical context, rather than on aneurysm exclusion alone. Observation is reserved for patients with typical features and no causative abnormality, whereas atypical or pupil-involving presentations with unrevealing imaging warrant specialist review and further targeted evaluation, including consideration of DSA if suspicion of a microaneurysm remains (18, 28, 34, 35). Because ischemic oculomotor palsies are not entirely benign (36), observation should be paired with vascular risk-factor control.

Once urgent structural and other serious causes have been excluded or treated, long-term rehabilitation should follow a systematic, timeline-based sequence to re-establish functional binocularity and appearance. Spontaneous recovery is common in ischemic cases within the first 12 weeks. For persistent deficits lasting past 6 months, carefully staged surgeries—where strabismus correction (ranging from standard recession-resection to multi-muscle transpositions and globe fixation) typically precedes ptosis repair—support individualized functional and esthetic outcomes while aiming to safeguard corneal health.

### 3.1 Limitations

As a narrative review designed to bridge the gap between acute neuro-ophthalmic triage and chronic ophthalmic rehabilitation, our methodology has inherent limitations. The expert-driven selection of literature introduces the potential for selection bias, and the exclusion of studies with fewer than five patients—while intended to ensure the statistical reliability of surgical outcomes—may omit highly specific case reports detailing rare neurovascular presentations. Furthermore, because this study does not adhere to systematic review protocols (e.g., PRISMA guidelines), a formal meta-analysis of surgical outcomes was not performed. The proposed framework has not been prospectively validated or endorsed by a guideline body; therefore, it is intended as an evidence-informed synthesis rather than a definitive standard of care.

### 3.2 Future directions

Although there have been substantial therapeutic advancements, the existing clinical literature still exhibits considerable gaps. Future research should focus on establishing prospective registries and multicenter observational cohorts with standardized outcome measures to systematically compare the long-term functional and esthetic outcomes of various advanced muscle transposition protocols. There is a pressing need to establish reliable predictive clinical models that can pinpoint patients with microvascular or partial TNP who are most likely to fail conservative therapy and monitoring. This would allow clinicians to streamline care and tailor surgical preparation timelines. Moving from fragmented specialty care toward a unified, bipartite roadmap may help shift TNP management from a period of diagnostic uncertainty to a more structured continuum of care. By sequencing acute neurovascular triage and chronic strabismus correction, this framework provides a structured approach that may facilitate timely management without sacrificing long-term visual rehabilitation.

## 4 Conclusion

This narrative review presents an evidence-informed, phase-based framework for evaluating and managing third nerve palsy. Early evaluation and appropriately directed neuroimaging are essential to identify aneurysmal and other potentially serious causes requiring urgent treatment. Following stabilization and etiological management, ophthalmic rehabilitation should be individualized according to the extent of spontaneous recovery, residual ocular misalignment, ptosis, diplopia, and patient priorities. While this framework integrates available evidence and clinical expertise, prospective studies are needed to evaluate its effect on diagnostic timeliness, visual function, quality of life, and clinical outcomes.
